# Acute viral hepatitis morbidity and mortality associated with hepatitis E virus infection: Uzbekistan surveillance data

**DOI:** 10.1186/1471-2334-9-35

**Published:** 2009-03-25

**Authors:** Makhmudkhan B Sharapov, Michael O Favorov, Tatiana L Yashina, Matthew S Brown, Gennady G Onischenko, Harold S Margolis, Terence L Chorba

**Affiliations:** 1 Tashkent Pediatric Medical Institute and Central Asia Epidemiology Network, Ministry of Health, Tashkent, Republic of Uzbekistan; 2 Division of Viral Hepatitis (World Health Organization Collaborating Center for Research and Reference in Viral Hepatitis), National Center for HIV/AIDS, Viral Hepatitis, STD and TB Prevention, Centers for Disease Control and Prevention, Atlanta, Georgia, USA; 3 Coordinating Office for Global Health, Centers for Disease Control and Prevention, Atlanta, Georgia, USA; 4 Division of Translational Research, International Vaccine Institute, Seoul, Korea; 5 Specialty Laboratories, Santa Monica, California, USA; 6 Federal Service for Surveillance on Consumer Rights and Wellbeing, Ministry of Health and Social Development of the Russian Federation, Moscow, Russia; 7 Pediatric Dengue Vaccine Initiative Program, International Vaccine Institute, Seoul, Korea; 8 Office of the Director, National Center for HIV/AIDS, Viral Hepatitis, STD, and TB Prevention, Centers for Disease Control and Prevention, Atlanta, Georgia, USA

## Abstract

**Background:**

In Uzbekistan, routine serologic testing has not been available to differentiate etiologies of acute viral hepatitis (AVH). To determine the age groups most affected by hepatitis E virus (HEV) during documented AVH epidemics, trends in AVH-associated mortality rate (MR) per 100,000 over a 15-year period and reported incidence of AVH over a 35-year period were examined.

**Methods:**

Reported AVH incidence data from 1971 to 2005 and AVH-associated mortality data from 1981 to 1995 were examined. Serologic markers for infection with hepatitis viruses A, B, D, and E were determined from a sample of hospitalized patients with AVH from an epidemic period (1987) and from a sample of pregnant women with AVH from a non-epidemic period (1992).

**Results:**

Two multi-year AVH outbreaks were identified: one during 1975–1976, and one during 1985–1987. During 1985–1987, AVH-associated MRs were 12.3–17.8 per 100,000 for the general population. Highest AVH-associated MRs occurred among children in the first 3 years of life (40–190 per 100,000) and among women aged 20–29 (15–21 per 100,000). During 1988–1995 when reported AVH morbidity was much lower in the general population, AVH-associated MRs were markedly lower among these same age groups. In 1988, AVH-associated MRs were higher in rural (21 per 100,000) than in urban (8 per 100,000) populations (RR 2.6; 95% CI 1.16–5.93; p < 0.05). Serologic evidence of acute HEV infection was found in 280 of 396 (71%) patients with AVH in 1987 and 12 of 99 (12%) pregnant patients with AVH in 1992.

**Conclusion:**

In the absence of the availability of confirmatory testing, inferences regarding probable hepatitis epidemic etiologies can sometimes be made using surveillance data, comparing AVH incidence with AVH-associated mortality with an eye to population-based viral hepatitis control measures. Data presented here implicate HEV as the probable etiology of high mortality observed in pregnant women and in children less than 3 years of age in Uzbekistan during 1985–1987. High mortality among pregnant women but not among children less than 3 years has been observed in previous descriptions of epidemic hepatitis E. The high mortality among younger children observed in an AVH outbreak associated with hepatitis E merits corroboration in future outbreaks.

## Background

In Uzbekistan, there is a high incidence of infection with hepatitis viruses including the viruses of hepatitis A (HAV) [1], hepatitis B (HBV) [2], hepatitis C (HCV) [3], hepatitis D (HDV) [4], and hepatitis E (HEV) [5]. In 1990–1995, of all reportable infectious processes, the number of reported cases of acute viral hepatitis (AVH) was exceeded only by the cumulative number of cases of acute respiratory disease [6]. AVH is a reportable disease in Uzbekistan, where periodic epidemics of fecal-orally-transmitted non-A, non-B hepatitis have been described [7,8]. Elsewhere, in regions in which epidemics of hepatitis E have been observed, increases in mortality among pregnant women have been reported [9-12].

For reporting purposes in Uzbekistan and other Central Asian republics of the former Soviet Union, the diagnosis of hepatitis A has generally been based on signs, symptoms and epidemiological data, while the diagnosis of acute hepatitis B has been based on serologic detection of hepatitis B surface antigen (HBsAg). However, lack of serological testing to differentiate hepatitis types has resulted in official statistics that have generally enumerated cases of AVH with typing of hepatitis A and hepatitis B only, and reporting of HCV, HDV, and HEV infections has not been routine.

An indicator of the impact of hepatitis infections is their associated mortality, most often the result of chronic liver disease [13]. However, few data exist concerning age- and gender-specific associations of AVH with mortality. We analyzed trends in AVH-associated mortality, including during periods in which hepatitis E epidemics have been documented [14,15], to determine age- and gender-specific changes in mortality.

## Methods

### Incidence Reporting

AVH incidence (morbidity) data from 1971 through 2005 were obtained from reports of the Sanitary-Epidemiologic Service (SES) of the Uzbekistan Ministry of Health (MOH). These are standardized data from a surveillance system that was uniformly used by the republics during the Soviet era [16,17] and that has continued to function in Uzbekistan since the dissolution of the Soviet Union.

Uzbekistan is organized into 12 provinces, called viloyats, and one autonomous republic; each of these is subdivided into administrative units called raions. At each level (republic, viloyat, raion), the SES has operated an infectious disease surveillance system in which AVH has been a reportable condition. The system has evolved under a mandate that requires all suspected or confirmed cases to be reported by telephone to the raion SES within 12 hours, followed by a written case report [17,18]. These reports are forwarded to the viloyat SES. Reporting sources include physicians and feldshers (primary health care providers with less formal training than physicians, found more commonly in rural areas) who evaluate patients in hospitals, polyclinics, diagnostic centers, ambulatory clinics, or feldsher stations (rural primary health care clinics). For each case, health care providers report patient's name, age, date of birth, sex, address, occupation, day care attendance, last day of work, polyclinic attended, primary diagnosis, date of symptom onset, date of physician visit, date of diagnosis, and date of hospitalization.

Telephone case reports received at the raion SES are recorded and updated as additional clinical, laboratory and epidemiologic information is received to help raion SES staff implement disease control measures. The raion SES obtains additional information on each case within 24 hours using a standardized case investigation form. Raion SES staff complete a separate case form which summarizes the information collected during the course of the epidemiologic and clinical work-up, including clinical presentation, vaccination status, and laboratory results. SES epidemiologists may request additional laboratory testing to confirm the healthcare provider's clinical diagnosis. However, lack of reagents to perform diagnostic testing and lack of strict case definitions have had the result that most reported cases of AVH in the SES database reflect clinical diagnoses that have not been laboratory confirmed.

Raions report AVH case totals to the viloyat SES, and raion cases are tabulated by age group (total, 0 to 14 years; <1 year (infancy); 1 and 2 years (up to but not including the third birthday); and 3 to 6 years). Annually, aggregated data are reported to the viloyat SES. Each month, the viloyat SES tabulates confirmed AVH cases by age group, and reports totals to the republican SES.

### Mortality Reporting

AVH-specific mortality data were examined as provided to the MOH by *Goskomstat*, the State Committee of Statistics of the Republic of Uzbekistan, a governmental vital records agency with its own raion-viloyat-republic reporting hierarchy that is separate from the SES. When a patient dies, the clinician notifies the hospital or polyclinic statistician, who in turn is responsible for reporting the death and its putative cause(s) to the statistical administrative units within *Goskomstat *that are concerned with mortality.

### Case Definition

Although there has been no strict case definition of AVH, MOH guidelines for clinicians have traditionally defined AVH as an infectious disease characterized by acute onset of jaundice, hepatomegaly, and (where available) a three-fold or greater elevation of serum aminotransferase levels. AVH is noted as a cause of death on a death certificate if a clinician believes the death was a complication of AVH.

### Laboratory Methods

During an identified AVH outbreak in 1987, serum samples were collected from a convenience sample of hospitalized patients with AVH in Eastern and Southern Uzbekistan (Fergana and Kashkadarya viloyats) on the first to seventh day after onset of jaundice; criterion for hospitalization was onset of jaundice. Similarly, during 1992, serum samples were collected from a convenience sample of hospitalized pregnant women with AVH in Tashkent viloyat on the first to seventh day after onset of jaundice; again, criterion for hospitalization was onset of jaundice. For both groups, case-patients were defined as those with acute onset of dyspepsia, jaundice, hepatomegaly, and a three-fold or greater increase in serum concentrations of alanine aminotrasferase. Serum specimens were delinked from personal identifiers at the time of collection and were stored at -20°C until subsequently tested. As both of these activities predated implementation of codified institutional accountability of human research protections, no standardized consenting process was employed; however, patient participation was limited to a solitary phlebotomy for minimal serum samples for diagnostic purposes.

Serologic testing by enzyme immunoassay (Abbott Laboratories, North Chicago, IL) for markers of infection with hepatitis viruses was performed at the Viral Hepatitis Diagnostic Department, Ivanovski Institute of Virology, Moscow. Testing included assays for markers of acute HAV infection (IgM anti-HAV); markers of acute HBV infection, HBsAg and IgM antibody to hepatitis B core antigen (IgM anti-HBc); and markers of acute HDV infection (IgM anti-HDV and total anti-HDV). Serum specimens which tested negative for all of the above markers were retained and subsequently tested in the Hepatitis Reference Laboratory, Division of Viral Hepatitis, National Center for Infectious Diseases, Centers for Disease Control and Prevention (CDC), for IgM and IgG antibody to HEV (IgM and IgG anti-HEV) using both Western blot and ELISA [19].

### Statistical Methods

#### Incidence data analyses

To quantify AVH case counts and rates per 100,000 population, we analyzed Republican SES reports for the period 1971–2005 and specific viloyat reports for 1985–1995. To verify reported rates by viloyat, population counts from viloyat reports were compared with official Uzbekistan census data.

#### Mortality data analyses

To ascertain mortality rates per 100,000 (MRs) from AVH, AVH-associated mortality data for 1981–1995 were obtained from the Department of Statistics of the Ministry of Health. AVH-associated MRs for the republic and for each viloyat for 1985–1995 were calculated by age and sex on the basis of mortality data analyzed from *Goskomstat *reports. Where mortality rates per 100,000 population were calculated for a specific age and sex group, denominator data were restricted to population estimates for that specific age and sex group. These analyses did not include deaths attributed to chronic viral hepatitis and/or cirrhosis.

### Statistical analyses

Data were analyzed using Epi Info 6.04 for relative risk (RR), Student's t-test, correlation analysis (r), Pearson's conformity test, or Fisher's exact test. Specifically, categorical data were analyzed using Pearson's χ^2^-test or Fisher's exact test, as appropriate; Fisher's exact test was used where categorical data were compared in which any cell size was <5. Continuous data were analyzed with Student's *t*-test. Statistical significance was determined by calculating a p value (with Yates' correction factor where needed) and exact 95% confidence intervals (CIs), or Taylor series 95% CIs, as appropriate. The significance level for all statistical analyses was p = 0.05.

Trends in AVH-associated mortality over the 15 years (1981–1995) for which aggregate data were available were compared with trends in reported AVH incidence over the same period using the Pearson product-moment correlation coefficient. For the 11-year period (1985–1995) for which gender- and age-specific AVH-associated mortality data were available, data were compared by gender, age, and rural/urban designation of the decedent using Pearson's χ^2^-test, Fisher's exact test, or Student's *t*-test as appropriate, depending on whether data were categorical or continuous.

In performing analyses of serology data, IgG anti-HEV data for various age groups of children were compared using Pearson's χ^2^-test.

## Results

### Reported Incidence

**Figure 1:**
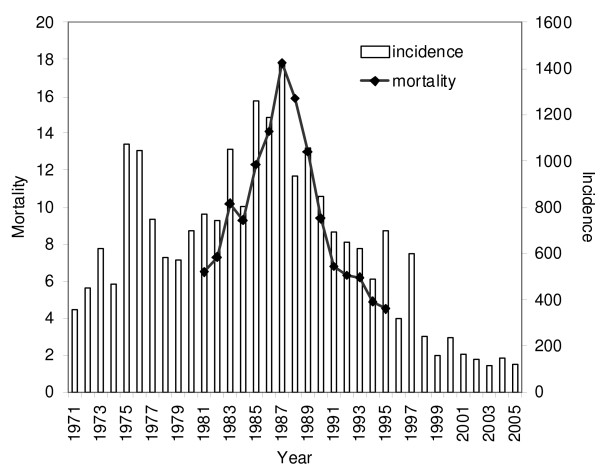
**Acute viral hepatitis incidence (1971–2005) and associated mortality rates (1981–1995), by year, Uzbekistan (per 100,000 population)**. Left ordinate (y-axis) and line graph: mortality per 100,000 population. Right ordinate (y-axis) and bar diagram: reported acute viral hepatitis incidence per population.

From 1971 through 2005, national reported AVH incidence in Uzbekistan was consistently greater than 200 cases per 100,000 (Fig. 1). Two multi-year epidemics of AVH were noted: one spanning the years 1975 and 1976 with annual incidences of 1047 and 1072 cases per 100,000, respectively, and another from 1985 through 1987 with annual incidences of 1259, 1187, and 1431 cases per 100,000. Single-year increases in AVH were also noted in 1973, 1983, 1995 and 1997, with peak incidences ranging from 600 to 1051 per 100,000.

Verification of reported population counts by viloyat revealed no discrepancies when viloyat reports for hepatitis rates were compared with official Uzbekistan census data. The geographic distribution of average annual AVH incidence varied: highest viloyat rates were reported in the East (Fergana, Namangan, and Andijan) and in the South (Kashkadarya, Surkhandarya), where incidence in some viloyats was 1.5- to 1.8-times the national average. In less densely populated areas, e.g., in the West (Karakalpakstan, Bukhara, and Navoi), incidence tended to be lower than the national average except in 1985–1987. Despite higher population density, reported AVH incidence in Tashkent was relatively low and exceeded the national rate only in 1995 and 1997.

### Reported Mortality

AVH-associated mortality varied over 1981 to 1995 and AVH-associated mortality was closely associated with reported AVH incidence, r = 0.92; p < 0.001 (Fig. 1). The highest nationwide AVH-associated mortality occurred in 1987 (17.8 per 100,000) with a corresponding AVH incidence rate of 1,431 per 100,000.

**Figure 2:**
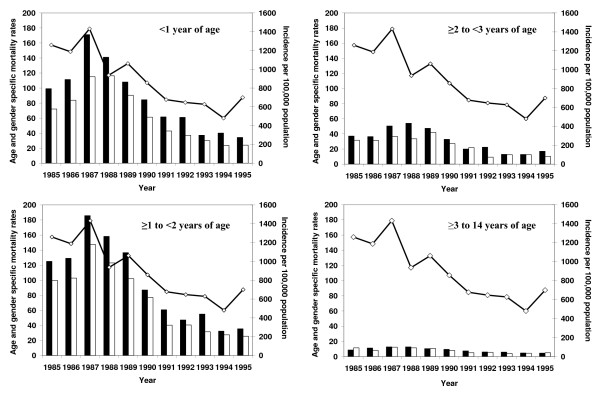
**Acute viral hepatitis-associated mortality rates for children in various age groups, and reported acute viral hepatitis incidence in all age groups, per 100,000 population, by year, Uzbekistan, 1985–1995**. Left ordinate (y-axis) and bar diagram: age- and gender-specific mortality: "black bars" – mortality among boys; "white bars" – mortality among girls, per 100,000 population of corresponding age and sex. Right ordinate (y-axis) and line diagram: reported acute viral hepatitis incidence in the general population, per 100,000.

In 1985–1995, no significant difference was observed between males and females: among males, the MR was 11.8 (95%CI: 10.3–13.3) per 100,000, and among females, the MR was 10.8 (95%CI: 9.3–12.3) (RR = 1.0; 95%CI: 0.8–1.4; p = not significant (n.s.)). When AVH-associated mortality was examined by age group for all ages, highest rates were observed in children aged <3 years (Fig. 2), especially among those aged <2 years; among both those aged <1 year and those aged ≥1 to <2 years, the peak coincided with the 1987 peak observed in reported AVH incidence. In Fig 2, gender-specific AVH-associated mortality for 1985–1995 by age group (<1 year, ≥1 to <2 years, ≥2 to <3 years, 3 to 14 years) is presented in a set of four bar diagrams. Because reported AVH incidence data were not available in the same age classifications as AVH-associated mortality, AVH incidence rate for all age groups combined is presented as a line diagram above each of the four bar diagrams to give the reader an appreciation of the temporal distribution of the AVH-associated age-specific mortality relative to the overall reported AVH morbidity. From 1987 to 1995, the AVH-associated MR decreased among infants (children aged <1 year) from 143.7 to 29.3 per 100,000 (p < 0.01), and among children aged ≥1 to <2 years from 166.8 to 30.6 per 100,000 (p < 0.01); markedly less impressive decreases in rates of AVH-associated mortality were observed among children aged ≥2 to <3 years and among those 3 to 14 years during this same period, reflecting at least in part the fact that older children had a much smaller risk of AVH-associated mortality at the height of the epidemic than did children aged <2 years.

From 1985 to 1995, significant gender differences in AVH-associated mortality among children were observed. Of 22,405 AVH-associated deaths, 11,793 deaths were among males, of whom 10,024 (85%) were aged 0–14 years; 10,612 deaths were among females, of whom 7121 (67.1%) were aged 0–14 years (RR [of death among males vs. females] = 1.27; 95% CI: 1.25–1.29, p < 0.001).

From 1985 to 1995, AVH-associated MRs ranged from 1.1 to 5.6 per 100,000 among males in different 5-year age groups aged 15 to 49 years, and from 2.6 to 7.8 per 100,000 among males aged >50 [see Additional file 1]. Marked AVH-associated mortality was noted among females aged 20–24 years and 25–29 years, with the highest rates in these age groups in 1986 (21.4 and 19.6 per 100,000, respectively) and 1987 (19.0 and 18.5 per 100,000, respectively).

During 1985–1987, AVH-associated MRs were 12.3–17.8 per 100,000 for the general population. In 1985–1989, AVH-associated MRs were consistently higher among females than among males in several age groups: 15–19 years; 20–24 years; 25–29 years; and 30–34-years [see Additional file 1]. In this same time period, among persons aged >40 years, females consistently had lower AVH-associated MRs than did males (1.8–3.5 vs. 2.7–6.9 per 100,000; p < 0.01). In 1986, among persons aged 20–24 years, AVH-associated mortality among females was more than 7 times that of males (21.4 vs. 2.9 per 100,000; p < 0.001) [see Additional file 1]. Among persons aged 15–19 years in 1993, there was an abrupt increase in AVH-associated mortality among females (11.7 per 100,000 females vs. 3.5 per 100,000 males) despite a decrease in the AVH incidence rate in the general population [see Additional file 1]. This dramatic rise in mortality was not observed in any other age group.

The geographic distribution of AVH-associated mortality varied: highest viloyat MRs were reported in 1987 in the Eastern viloyats (Fergana, Namangan, and Andijan: range 20.1 to 31.0 per 100,000) and in the South (Kashkadarya, and Surkhandarya: range 23.0 to 25.0 per 100,000). From 1985 through 1995, AVH-associated MRs were higher in rural rather than in urban populations, with decreasing trends in both populations: 21.0/8.0 (RR= 2.6; 95% CI: 1.16–5.93, p < 0.05) per 100,000 rural/urban population in 1988; 18.0/6.5 (RR = 2.6; 95% CI: 1.07–6.16, p < 0.05) in 1989; 12.4/4.8 (RR = 2.4; 95% CI: 0.85–6.81, p = n.s.) in 1990; 9.3/7.0 (RR = 1.3; 95% CI: 0.48–3.45, p = n.s.) in 1991; 8.4/3.8 (RR = 2; 95% CI: 0.6–6.64, p = n.s.) in 1992 and 3.4/3.2 (RR = 1.1; 95% CI: 0.2–4.95, p = n.s.) in 1993.

### HEV Serology

Of 396 adults and children with AVH for whom serum samples were available from the 1987 epidemic in southern and eastern Uzbekistan (Kashkadarya and Fergana viloyats), 280 (70.7%) were IgG anti-HEV positive; of these, 221 (78.9%) were also IgM anti-HEV positive. Among children with AVH, IgG anti-HEV was detected in 18/58 (31.0%) of those aged <3 years, 22/32 (68.7%) of those aged 3–6 years, 21/37 (56.7%) of those aged 7–10 years, and 20/35 (57.1%) of those aged 11–14 years (p = n.s.). The highest anti-HEV IgG prevalence was found among those aged 20–29 years (104/122; 85.2%); and 30–39 years (52/63; 82.5%). Evidence of acute HAV infection (i.e., IgM anti-HAV) was found in 78 of the 396 (19.7%) and acute HBV infection (i.e., IgM anti-HBc) in 33 of the 396 (8.3%); acute HDV infection (i.e., anti-HDV IgM and anti-HDV) in 3 (0.8%), and acute mixed infection with HAV and HBV in 2 (0.5%); none of these was IgM anti-HEV positive.

Of 99 pregnant women with AVH for whom serum samples were available from 1992 from Tashkent viloyat, 12 (12%) tested positive for IgM and IgG anti-HEV, with one AVH-associated death (8%; 95% CI 4–40); 55 (55%) had evidence of acute HBV infection, with one AVH-associated death (2%; 95% CI 0.1–10); 16 had acute HDV superinfection, and two of these had AVH-associated deaths (12.5%; 95% CI 2–40).

## Discussion

This study documented sustained high AVH incidence over a 35-year period in Uzbekistan and two large AVH epidemics, the latter of which was associated with documented acute HEV infection in serum specimens gathered from AVH cases hospitalized during the epidemic. During both of these epidemics, a dramatic increase in AVH-associated mortality was observed among females 15–39 years of age, and in the first of these epidemics, among children <3 years of age. Among children, reported AVH-associated mortality was significantly higher among boys than among girls. Reported AVH incidence and AVH-associated mortality during these epidemics were 2–3 times higher in rural areas than in urban areas. As these epidemics waned, the mortality rate for women of childbearing age decreased dramatically. Similar hepatitis E epidemics have also been documented in adjacent, less-densely populated republics, – in 1983–1984 in Turkmenistan immediately to the West [5], and in 1986–1987 in Tajikistan and Kyrgyzstan immediately to the East [20].

In 1993, the rise in AVH-associated mortality in Uzbekistan among women was relatively limited to those aged 15-19 years; from the serologic data that we have presented and from the fact that the mortality was confined to women of childbearing age, we posit that these data reflected a hepatitis E outbreak relatively limited to persons under 20 years of age. In this case, the distribution of ages affected may have reflected a cohort effect of HEV immunity among older women of childbearing age who were exposed in the 1985–1987 epidemics. In the more recent years of this analysis, there was a marked decrease in mortality among women aged 20–29 years associated with the overall decrease in AVH incidence and mortality. During 1991-1995, when AVH was not epidemic across the whole population, the AVH-associated MR among women aged 20-24 and 25-29 years ranged from 2.6 to 7.3 per 100,000 women. Based on these data, it appears that HEV infection was a large contributor to the AVH-associated deaths during this period, most likely among young pregnant women. In the absence of laboratory confirmation, AVH-associated mortality among reproductive age women may be of use as an indicator of HEV infection.

Although the patterns of mortality observed among women of childbearing age were consistent with observations from previous descriptions of hepatitis E epidemics [5,7,9-11], we have not identified previous population-based reports of increased mortality among young children in association with HEV infection. However, from these and other endemic areas, it has been reported that younger children are more likely to have more severe disease than do persons in other age groups if infected with HEV [21-27]. Although infant MRs before the epidemic period that began in 1985 had been similar to those found elsewhere on average in the former Soviet Union, AVH-associated mortality in children under the age of 2 years in Uzbekistan was five times that observed on average across the former Soviet Union during this epidemic period [13].

In reviewing the morbidity and mortality data presented here, other causes of AVH were considered. The dramatic increase in AVH-associated mortality in the youngest age groups during the HEV epidemic period could not be ascribed solely to either of the other two most common causes of acute liver failure in the pediatric population in this region, namely HAV infection or HBV infection. HAV infection in young children in a highly endemic area is almost always asymptomatic and rarely results in death [8,13,28-30]. As for acute HBV infection, over the period 1985–1995, there was little fluctuation in documented disease incidence in the youngest age groups in Uzbekistan [6]. However, it is possible that the increase in AVH-associated mortality observed in the youngest age groups at least in part reflected HEV infection accompanied by another wide-spread cause of acute liver failure, e.g., HAV or HBV co-infection, drug-induced liver failure, or an environmental toxin. Indeed, it has been observed that children with HAV and HEV co-infection have a higher incidence of acute liver failure than do children who do not have co-infection [31]. Both hepatotoxic mycotoxin contamination of grain-based foodstuffs [32] and hepatoxic pyrrolizidine-alkaloid ingestion as the result of *Heliotropium lasocarpium *contamination in wheat production [33-35] are well-described phenomena in the region; and at least one case of hepatitis associated with IgM anti-HEV antibodies has been described in a child, developing after starting dapsone and resolving after discontinuing the medication, suggesting drug-induced potentiation of hepatitis E [36]. However, no known AVH etiology other than HEV was widely observed or suspected among children in this region during 1985 through 1987. Although the high mortality rate observed in this outbreak among children under the age of 3 years, especially those under the age of 2 years, suggests that this group may be at greater risk for developing more severe forms of AVH in a epidemic of hepatitis E, mortality case-specific serology data were not available to determine whether any other cause of acute liver failure may have contributed to the increase in mortality.

In 1985 to 1987, when AVH mortality was greatest, there was a mortality increase among those aged between 1 and 3 years that was much greater than that among infants (children less than 1 year of age). Understandably, those aged between 1 and 3 years generally would have a greater exposure than infants because of the increased likelihood of using water sources rather than continuing breast-feeding and because of greater mobility and access of the child to sources of HEV exposure. Infections from contaminated food and water begin after breastfed babies are weaned [37].

Protective factors in breast milk and maternal antibodies could also explain why infants fared better than children aged between 1 and 3 years in an environment with HEV exposure: IgG antibodies cross the placenta well during the third trimester [38], and secretory IgA antibodies and other host defense factors in breast milk, such as lactoferrin, lysozyme, and macrophages and granulocytes, can be protective or mitigate infection [37,39]. Milk-excreted antibodies play a role in protecting infants from infection by pathogens having a mucosal portal of entry, such as rotavirus [40,41]. However, in the 1985–1987 outbreak described here, only 8–10% of all healthy women had anti-HEV IgG in serum (data not shown), so one would expect that only 8–10% of infants would have had maternal antibody protection from HEV infection. In evaluating future outbreaks, if only a small fraction of infants has antibodies to HEV and if increases in hepatitis E-associated mortality observed among children between 1 and 3 years of age are greater than those observed among infants, alternative hypotheses will need to be considered to explain this latter phenomenon.

## Conclusion

Because morbidity and mortality from AVH are the result of social, economic and environmental factors, the effectiveness of population-based viral hepatitis control measures depends on targeted, etiology-focused efforts including implementation of virus-specific surveillance. In the case of hepatitis E, the goal of such efforts is to improve hygiene and to assist in determining potential target populations for a highly efficacious, recombinant vaccine that has been developed and tested but is not yet available [42,43]. In Uzbekistan, surveillance based exclusively on clinical and epidemiologic data has been inadequate; the only test routinely available for viral hepatitis has been identification of HBsAg, with no other test readily available to differentiate viral hepatitis types. Because studies of AVH mortality in the former Soviet Union have lacked accurate etiology-specific data [13], morbidity statistics for specific viral hepatitis diagnoses have been unreliable. However, our findings suggest that when AVH incidence is compared with AVH-associated mortality, it may be logical to make some inferences regarding probable hepatitis etiologies. Ecological studies such as this can serve to generate hypotheses for more definitive studies.

In addition to the known increased mortality among young women of child-bearing age in association with an epidemic of hepatitis E, the increased mortality observed here among children up to 3 years of age merits evaluation and corroboration in future outbreaks.

## Competing interests

The authors declare that they have no competing interests.

## Authors' contributions

MBS collected statistical data, made the preliminary data analyses, and drafted the manuscript. MOF conceived and designed the study, supervised data collection and coordination, and conducted quality control/quality assessment of laboratory testing of specimens for IgM and IgG antibody to HEV (IgM and IgG anti-HEV). TLY and GGO supervised gathering of specimens and conducted laboratory testing including assays for markers of acute HAV infection (IgM anti-HAV), field laboratory testing of specimens for IgM and IgG antibody to HEV (IgM and IgG anti-HEV); markers of acute HBV infection, HBsAg and IgM antibody to hepatitis B core antigen (IgM anti-HBc); and markers of acute HDV infection (IgM anti-HDV and total anti-HDV). MSB participated in design of the study and performed statistical analysis. HSM participated in development of discussion and supervised study efforts. TLC developed the design of the article, and contributed substantially to description of methods, data collection, and discussion of results. All authors read and approved the final manuscript.

## Pre-publication history

The pre-publication history for this paper can be accessed here:

http://www.biomedcentral.com/1471-2334/9/35/prepub

## Supplementary Material

Additional file 1 **Acute viral hepatitis associated mortality rates for various age groups, by sex and year, Uzbekistan, 1985–1995 (per 100,000 population of corresponding age and sex).** The data provided represent acute viral hepatitis-associated mortality rates per 100,000 population of corresponding age and sex, for various age groups, by sex and year, based on death certificate data in Uzbekistan, 1985–1995. Click here for file
