# Supplementary material for: Acute viral hepatitis morbidity and mortality associated with hepatitis E virus infection: Uzbekistan surveillance data
Source: BMC Infect Dis. 2009 Mar 25;9:35. doi: 10.1186/1471-2334-9-35 (PMC2671511; doi:10.1186/1471-2334-9-35)
Supplement: Additional file 1 — Acute viral hepatitis associated mortality rates for various age groups, by sex and year, Uzbekistan, 1985–1995 (per 100,000 population of corresponding age and sex). The data provided represent acute viral hepatitis-associated mortality rates per 100,000 population of corresponding age and sex, for various age groups, by sex and year, based on death certificate data in Uzbekistan, 1985–1995. [file 1471-2334-9-35-S1.doc]

**Acute viral hepatitis associated mortality rates for various age groups (per 100,000 population of corresponding age and sex), by sex and year, Uzbekistan, 1985-1995.**

| **Age group (years)** | **1985** | | **1986** | | **1987** | | **1988** | | **1989** | | **1990** | | **1991** | | **1992** | | **1993** | | **1994** | | **1995** | |
| --- | --- | --- | --- | --- | --- | --- | --- | --- | --- | --- | --- | --- | --- | --- | --- | --- | --- | --- | --- | --- | --- | --- |
|  | **Male** | **Female** | **Male** | **Female** | **Male** | **Female** | **Male** | **Female** | **Male** | **Female** | **Male** | **Female** | **Male** | **Female** | **Male** | **Female** | **Male** | **Female** | **Male** | **Female** | **Male** | **Female** |
| 15-19 | 1.7 | 3.9 | 3.1 | 4.4 | 2.9 | 4.0 | 2.8 | 3.7 | 4.4 | 4.7 | 3.9 | 3.8 | 2.3 | 2.5 | 2.3 | 2.7 | 3.5 | 11.7 | 4.0 | 3.1 | 3.0 | 1.5 |
| 20-24 | 2.4 | 18.4 | 2.9 | 21.4 | 3.4 | 19.0 | 2.4 | 13.2 | 2.5 | 11.1 | 4.4 | 2.0 | 2.3 | 6.1 | 1.9 | 7.3 | 3.2 | 4.5 | 1.7 | 5.2 | 1.6 | 4.7 |
| 25-29 | 2.9 | 14.7 | 4.5 | 19.6 | 5.6 | 18.5 | 3.7 | 12.0 | 2.7 | 8.2 | 3.7 | 1.5 | 1.6 | 4.1 | 2.2 | 5.5 | 2.7 | 4.6 | 2.2 | 2.6 | 1.7 | 3.1 |
| 30-34 | 1.8 | 8.0 | 2.4 | 10.7 | 3.0 | 9.8 | 3.2 | 6.9 | 1.3 | 4.6 | 2.9 | 1.7 | 1.9 | 3.0 | 1.6 | 3.4 | 2.2 | 2.3 | 1.2 | 2.2 | 1.6 | 2.3 |
| 35-39 | 2.4 | 3.4 | 2.8 | 7.0 | 1.1 | 5.4 | 1.8 | 4.0 | 3.1 | 2.5 | 2.0 | 1.8 | 1.3 | 2.1 | 1.3 | 1.8 | 1.7 | 2.9 | 1.6 | 1.2 | 1.4 | 1.4 |
| 40-49 | 2.9 | 2.0 | 3.1 | 2.5 | 2.5 | 2.3 | 3.3 | 2.0 | 3.5 | 2.3 | 1.6 | 1.0 | 2.5 | 2.4 | 1.6 | 1.6 | 1.7 | 1.3 | 1.2 | 1.2 | 1.4 | 1.1 |
| 50-59 | 2.7 | 2.6 | 4.0 | 2.1 | 4.5 | 2.5 | 4.0 | 2.4 | 3.9 | 3.4 | 4.0 | 1.6 | 3.0 | 2.3 | 2.8 | 2.0 | 4.0 | 1.7 | 4.2 | 3.5 | 2.6 | 2.3 |
| > 60 | 4.2 | 1.8 | 4.2 | 1.9 | 3.7 | 2.5 | 6.9 | 2.6 | 5.6 | 3.5 | 5.7 | 2.0 | 2.6 | 2.8 | 5.6 | 3.3 | 7.8 | 4.9 | 7.4 | 4.2 | 5.7 | 4.8 |
